# Small for gestational age is associated with reduced lung function in middle age: A prospective study from first to fifth decade of life

**DOI:** 10.1111/resp.14379

**Published:** 2022-10-05

**Authors:** Melvin Tandra, E. Haydn Walters, Jennifer Perret, Adrian J. Lowe, Caroline J. Lodge, David P. Johns, Paul S. Thomas, Gayan Bowatte, Peter G. Davis, Michael J. Abramson, Shyamali C. Dharmage, Dinh S. Bui

**Affiliations:** ^1^ Allergy and Lung Health Unit, School of Population and Global health The University of Melbourne Melbourne Victoria Australia; ^2^ School of Medicine and Menzies Institute University of Tasmania Hobart Tasmania Australia; ^3^ Inflammation and Infection Research, Faculty of Medicine University of New South Wales Sydney New South Wales Australia; ^4^ Department of Basic Sciences, Faculty of Allied Health Sciences University of Peradeniya Peradeniya Sri Lanka; ^5^ Department of Obstetrics and Gynaecology University of Melbourne Melbourne Victoria Australia; ^6^ Department of Newborn Research The Royal Women's Hospital Melbourne Victoria Australia; ^7^ School of Public Health & Preventive Medicine Monash University Melbourne Victoria Australia

**Keywords:** adult lung function deficit, low birth weight, restriction, small for gestational age, spirometry, Tasmanian Longitudinal Health Study

## Abstract

**Background and Objective:**

The association between birth weight, particularly relative to gestational age, and adult lung function is uncertain. We investigated the associations between birth weight relative to gestational age and measures of lung function in middle age, and mediation of these associations by adult height.

**Methods:**

Participants in the Tasmanian Longitudinal Health Study who had both known birth weight and lung function assessment at age 45 years were included (*n* = 849). Linear regression models were fitted to investigate the association between small for gestational age and birth weight with post‐bronchodilator lung function measures (forced expiratory volume in 1 second [FEV_1_], forced vital capacity [FVC], FEV_1_/FVC, diffusing capacity for carbon monoxide [D_L_co], residual volume [RV] and total lung capacity [TLC]), adjusting for potential confounders. The contribution of adult height as a mediator of these associations was investigated.

**Results:**

Compared with infants born with normal weight for gestational age, those born small for gestational age had reduced FEV_1_ (coefficient: −191 ml [95%CI: −296, −87]), FVC (−205 ml [−330, −81]), TLC (−292 ml [−492, −92]), RV (−126 ml [−253, 0]) and D_L_co (−0.42 mmol/min/kPa [−0.79, −0.041]) at age 45 years. However, they had comparable FEV_1_/FVC. For every 1 kg increase in birth weight, lung function indices increased by an average of 117 ml (95%CI: 40, 196) for FEV_1_, 124 ml (30, 218) for FVC, 215 ml (66, 365) for TLC and 0.36 mmol/min/kPa (0.11, 0.62) for D_L_co, independent of gestational age, but again not for FEV_1_/FVC. These associations were significantly mediated by adult height (56%–90%).

**Conclusion:**

Small for gestational age was associated with reduced lung function that is likely due to smaller lungs with little evidence of any specific parenchymal impairment.

## Graphical abstract

Our study provides new evidence that small for gestational age is associated with reduced lung function consistent with a restrictive pattern in middle age. This suggests that the specific long‐term effect of reduced fetal lung growth relates to restrictive lung function pattern that is likely due to smaller lungs.

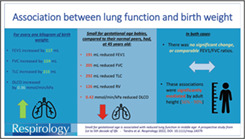

**See related** Editorial

## INTRODUCTION

The adverse impacts of small for gestational age (SGA) and low birth weight impose a considerable public health burden worldwide. Globally, the prevalence of low birth weight was estimated to be 14.6% (20.5 million babies) in 2015.^1^ The prevalence of SGA was estimated to be even higher, particularly in low‐income and middle‐income countries (up to 27%).^2^ Low birth weight is associated with increased mortality during infancy, childhood and adolescence.^3^ Moreover, low birth weight may be related to lifelong adverse health impacts including neurological, gastrointestinal and respiratory problems.^4^ Both animal and human studies suggest that low birth weight is associated with structural and functional changes in lung development.^5^

There is some evidence for associations of SGA and low birth weight with decreased spirometric indices in childhood and adolescence.^6^, ^7^, ^8^, ^9^, ^10^ Notably, one study has reported an association between SGA and reduced FVC in mid‐thirties.^11^ Similarly, studies have found positive associations between birth weight and FEV_1_ and FVC in adults.^12^, ^13^ While these studies support associations between SGA and lower birth weight and spirometric restriction (reduced FVC and FEV_1_ but preserved FEV_1_/FVC) in adults, none have examined more complex measures of static lung volumes including residual volume (RV) or total lung capacity (TLC) to confirm true lung restriction. Furthermore, post‐bronchodilator spirometry measures which are needed to quantify fixed airflow obstruction, have not been previously reported.

The potential associations of SGA and low birth weight with adult lung function deficits may involve different direct and indirect pathways. SGA and low birth weight are associated with lower lung function at birth and this may persist into later life.^14^ SGA and low birth weight are also associated with reduced somatic growth, potentially resulting in suboptimal height in adults, which would itself be a cause of reduced absolute lung function values in adulthood. Thus, adult height is a potential mediator of the associations between SGA and low birth weight and adult lung function deficits. Untangling the pathways between SGA and low birth weight and adult lung function deficits may inform clinical and public health interventions. However, the extent of this mediation effect has not previously been examined.

In this study, we aimed to: (1) investigate the association between SGA, lower birth weight and lung function including complex lung function measures in middle age, and (2) assess the role of adult height as a mediator of these associations.

## METHODS

### Study design and population

We used data from the Tasmanian Longitudinal Health Study (TAHS).^15^ The TAHS started in 1968 and recruited 98.8% children born in 1961 and attending school in Tasmania (*n* = 8583), in which parents completed a respiratory questionnaire and children attended a clinical examination. Participants were followed in 1974, 1979 and 1991. A subsequent follow‐up in 2002 included a postal survey of all successfully traced participants (*n* = 7562, 88.1%) with a response rate of 78.4% (*n* = 5729), from the original 1968 cohort. A subgroup of these respondents, enriched for cases of asthma or those with cough in childhood and adulthood, was invited to a more detailed laboratory study (*n* = 2387), of whom 1405 (58.9%, mean age 45 years) took part in the laboratory visit including lung function testing. Parents were also followed in 2010.

Data on birthweight and gestational age were extracted from birth records (Appendix S1 in the Supporting Information). We obtained all available hospital birth records for children born in 1961 in Tasmania from the Tasmanian State Archive and from Tasmanian hospitals (2775 participants). Parents provided birth weight information for 2265 participants, of whom 859 also had birth weight from hospital records. Thus, 4181 participants had information on birth weight (2775 from hospital records and additional 1406 from parental report). Among those who had birth weight data from both hospital records and parents record (*n* = 859), the mean (SD) difference between the two sources was 39.4 (228)g. At 45 years, lung function tests, including spirometry (FEV_1_, FVC, FEV_1_/FVC), single breath diffusing capacity (D_L_co, D_L_co/V_A_) and lung volumes (RV, TLC), were measured according to the joint American Thoracic Society and European Respiratory Society criteria^16^, ^17^, ^18^ (*n* = 1389). Spirometry was repeated 10 min after 300 μg of salbutamol was administered via a spacer. The 849 participants with both birth weight data and lung function measurements at 45 years formed the sample for this analysis.

### Definition of variables

SGA was defined as having a birth weight lower than the 10th percentile for a given gestational age. The 10th percentile cut‐off was taken from a previously published study on gestational ages and birth weights of Australian children.^19^ Low birth weight was defined by the WHO criteria as a birth weight below 2.5 kg. Height and weight were measured again at 45 years. Parental smoking status during pregnancy was obtained by self‐report. Early life socioeconomic status (SES) was based on the father's occupation.

### Statistical analysis

Descriptive statistics were produced and comparisons made using *t*‐tests or chi‐square tests where appropriate. The associations between exposures of interest (SGA as a binary variable and birth weight as a continuous variable) and post‐bronchodilator (post‐BD) lung function measures were examined using multiple linear regression. Directed acyclic graphs (DAGs, Figure S1 in the Supporting Information) were constructed to select the minimum set of confounders including sex, parental smoking, parental SES, mother's age at birth and parity. Gestational age was additionally adjusted for when the outcome was birth weight. Potential effect modification by sex, preterm‐birth, weight status at 45 years and personal smoking was also tested using likelihood ratio tests. Personal smoking was not included in the final model as a covariate because it was not considered as a potential confounder given it occurred after the exposure of interest.

As height at 45 years was determined as a mediator in the causal pathway from SGA to adult lung function (Figure S1 in the Supporting Information), we used absolute lung function values as the outcome, in order to assess the total effect of SGA on lung function outcomes. Then, a formal mediation analysis was performed to investigate the possibility and degree of the mediation effect by height.^20^, ^21^ Mediation analysis was performed for lung function indices that were significantly associated with the exposures.

Data analysis was performed using Stata (version 15.1; Stata Corp, College Station, TX).

## RESULTS

### Characteristics of participants

Table 1 summarizes the characteristics of 849 participants who had data on both birth weight and lung function available at 45 years (mean ± SD age 44.6 ± 0.85). Just over half of the participants were male (53.6%). Participants had a mean birth weight of 3.32 ± 0.59 kg, 18.1% of participants were born SGA and 11.3% born preterm. The rates of paternal and maternal smoking during pregnancy were 62.8% and 18.7%, respectively. At 45 years, participants had mean BMI 28.4 ± 6.1 kg/m^2^ and 27% were current smokers. Participants had mean post‐BD %predicted FEV_1_ of 97.9 ± 13.1% and %predicted FVC of 100.2 ± 11.9% at 45 years.

**TABLE 1:** Characteristics of participants (*n* = 849)

Characteristics	
Male sex, %	53.6%
Age of participants, mean (SD) years	44.6 (0.84)
Smoking status	
Never	42.7%
Past	30.3%
Current	27.0%
BMI at 45 years, mean (SD) kg/m^2^	28.4 (6.1)
Current asthma, %	23.5%
Birthweight, mean (SD) kg	3.32 (0.59)
Gestational age, mean (SD) weeks	39.5 (1.8)
Low birth weight, %	6.1%
Small for gestational age, %	18.1%
Maternal smoking during pregnancy, %	20.3%
Paternal smoking during pregnancy, %	62.8%
Maternal age at birth, mean (SD) years	27.4 (7.5)

Those missing birth weight data had similar baseline characteristics including maternal age at birth, socio‐economic status, asthma, pneumonia, height and lung function at 7 years compared with those with data available on birth weight (Table S1 in the Supporting Information). Those with lung function data available at 45 years had similar birth weight to those without lung function data (3.32 ± 0.59 kg and 3.34 ± 0.55 kg, respectively).

Those with and without SGA were similar in sex distribution, smoking status, BMI, adult asthma, maternal age at birth and parental smoking exposure. However, those with SGA had significantly lower adult height and lung function values including FEV_1_, FVC and TLC at 45 years (Table S2 in the Supporting Information). Those with SGA also had slightly lower childhood height (118.9 ± 5.1 vs. 120.8 ± 6.3 cm, mean difference = 1.8 cm, 95%CI [0.73, 3.0]) and this difference was slightly higher for adulthood height (168.4 ± 8.0 vs. 171.2 ± 8.5 cm, mean difference = 2.7 cm, 95%CI [1.2, 4.3]) compared to those without SGA.

### Association between SGA and birth weight and lung function at 45 years

Compared with middle‐aged participants who had normal birthweight for gestational age, SGA was significantly associated with reduced post‐BD FEV_1_ (mean difference −191 ml [95%CI: −296 to −87]) and FVC (mean difference −205 ml [95%CI: −330 to −81]) with normal FEV_1_/FVC (Table 2). SGA was also associated with reduced TLC (mean difference −292 ml [95%CI: −492 to −92]), RV (mean difference −126 ml [95%CI: −253, 0]) and D_L_co (mean difference −0.42 mmol/min/kPa [95%CI: −0.79 to −0.041]). Similar trends were seen for the associations between SGA and reduced FRC, but no association was seen with transfer coefficient of the lung for carbon monoxide (K_CO_).

**TABLE 2:** Adjusted associations between SGA and birth weight and post‐bronchodilator lung function at 45 years

	Small for gestational age (binary variable)	Birthweight adjusted for gestational age
	Mean difference (95%CI)^a^	Mean difference (95%CI) per kg^b^
FEV_1_, ml	−191 (−296 to −87)***	117 (40 to 196)**
FVC, ml	−205 (−330 to −81)***	124 (30 to 218)*
FEV_1_/FVC ratio, %	−0.41 (−2.01 to 1.19)	0.17 (−0.92 to 1.27)
TLC, ml	−292 (−492 to −92)**	215 (66 to 365)**
RV, ml	−126 (−253 to 0)	45 (−40 to 130)
D_L_co, mmol/min/kPa^c^	−0.42 (−0.79 to −0.041)*	0.36 (0.11 to 0.62)**
K_CO_, mmol/min/kPa/L	−0.019 (−0.075 to 0.036)	0.008 (−0.029 to 0.046)
FRC, ml	−153 (−312 to 4.8)	86 (−26 to 198)

*Note*: *p < 0.05; **p < 0.01; ***p < 0.001.

^a^ Adjusted for sex, age, age of mother at birth, parental smoking, number of siblings and parental social class.

^b^ Adjusted for sex, age, age of mother at birth, parental smoking, number of siblings, parental social class, as well as gestational age.

^c^ Values were adjusted to a standard haemoglobin concentration and corrected for the presence of carboxyhaemoglobin.

Birth weight was positively associated with post‐BD FEV_1_ (mean difference 117 ml [95%CI: 40–196] per kg increase in birth weight), FVC (mean difference 124 ml, [95%CI: 30–218] per kg increase in birth weight), but not with FEV_1_/FVC, adjusted for gestational age (Table 2). Birth weight was also positively associated with TLC (mean difference 215 ml, [95%CI: 66–365] per kg increase in birth weight) and D_L_co (mean difference 0.36 mmol/min/kPa, [95%CI: 0.11–0.62] per kg increase in birth weight), but again not with K_CO_.

There was no evidence of interaction of birth weight with sex, pre‐term birth, adult smoking or adult weight on lung function at 45 years (all *p*‐values > 0.1). Findings for pre‐BD spirometry measures were similar to those for post‐BD spirometry measures (Table S3 in the Supporting Information). When only birth weight data from hospital records were used in the sensitivity analysis, findings for the association between birth weight and lung function were similar (Table S4 in the Supporting Information). In an additional analysis when *z*‐scores of spirometry and lung volume indices were investigated as the outcomes, associations had the same direction but became non‐significant (Table S5 in the Supporting Information).

### Formal mediation analysis for the indirect effect by height

The associations of SGA and birth weight with FEV_1_ were mediated 58.0% and 65.9% by adult height (at 45 years), respectively (Table 3). The associations for FVC were mediated 86.5% and 90.0%, respectively. Similarly, height mediated 78.5% and 80.6% of the associations of SGA and birth weight with TLC. The corresponding outcomes for D_L_co were 66.8% and 55.8%, respectively. We conducted additional mediation analysis investigating childhood height (at 7 years) as a mediator. While we observed possible evidence of mediation by childhood height, the magnitude of this mediation was substantially smaller than that through adult height (Table S6 in the Supporting Information). This suggested that attained adult height (rather than childhood height) played an important role in the pathway between SGA and adult lung function.

**TABLE 3:** Mediation analysis for height as a mediator of the associations between birth weight and small for gestational age with lung function at 45 years

Outcomes	Indirect effect (ACME) (95%CI)	Direct effect (95%CI)	% mediated
Association with small for gestational age			
FEV_1_	−0.114 (−0.165 to −0.066)	−0.083 (−0.183 to 0.026)	58.0%
FVC	−0.183 (−0.257 to −0.108)	−0.030 (−0.143 to 0.094)	86.5%
TLC	−0.262 (−0.377 to −0.145)	−0.074 (−0.250 to 0.120)	78.5%
D_L_co	−0.294 (−0.449 to −0.154)	−0.140 (−0.474 to 0.228)	66.8%
Association with birth weight			
FEV_1_	0.078 (0.044 to 0.115)	0.040 (−0.037 to 0.114)	65.9%
FVC	0.124 (0.074 to 0.176)	0.014 (−0.007 to 0.042)	90.0%
TLC	0.169 (0.092 to 0.248)	0.041 (−0.094 to 0.170)	80.6%
D_L_co	0.195 (0.100 to 0.294)	0.161 (−0.095 to 0.407)	55.8%

Abbreviation: ACME, average causal mediation effects.

## DISCUSSION

While previous studies suggest a link between SGA and low birth weight and reduced FVC,^12^ ours provides insights by comprehensively investigating more complex lung function measures that included static lung volumes in middle aged participants from a longitudinal cohort. We found SGA to be associated with lower FEV_1_, FVC, TLC, RV and D_L_co levels in middle age. While reduced FVC can also occur in airway obstruction due to air trapping and high RV, it is notable that we had the benefit of data on static lung volumes to untangle and confirm the specific type of impact of SGA (on lung restriction rather than airway obstruction). Our study also disentangled the role of achieved height as a significant mediator on the causal pathway between SGA and reduced adult lung function.

SGA was associated with reduction in absolute lung function and these associations were largely mediated by adult height. However, associations with *z*‐scores of lung function were non‐significant, which is consistent with the findings from mediation analysis. Together, these findings suggest that such reduction in adult lung function associated with SGA is most likely related to smaller lungs, which is not necessarily a ‘disease’ in itself.

Restrictive lung deficits have been associated with increased respiratory symptoms, adverse cardiovascular outcomes and functional limitations, as well as mortality.^22^ Studies have shown that restrictive deficits are as prevalent as obstructive deficits in the general population and highlighted the need for early preventive strategies.^23^ Our findings revealed SGA to be a potentially modifiable risk factor for restrictive lung deficits. The findings may be helpful in primary care, allowing identification of at‐risk groups for early treatment, risk management or most importantly, prevention, of poor adult lung function. Moreover, our findings raise the possibility that maximum height mediates the association between SGA and adult lung function, and therefore it can be targeted to modify the adverse lung function outcomes related to SGA. However, we cannot firmly conclude that improvement in height would reduce the adverse effects of SGA on lung function because these are observational data and intervention trials to maximize attained adult height in SGA infants would be required to establish causation.

SGA is a proxy of reduced fetal growth, which may be associated with genetic factors and/or adverse intrauterine exposures. Several factors, including maternal smoking, low SES and poor maternal diet during pregnancy, are associated with reduced fetal growth and low birth weight.^24^, ^25^ However, it is likely that other underlying factors remain unknown. In this study, we were able to adjust for potential confounding by SES and parental smoking during pregnancy and gestational age. Our findings are consistent with the ‘fetal origins’ hypothesis also known as the ‘Barker hypothesis’, which proposed that impaired intrauterine growth may lead to long‐term consequences for many aspects of adult life, including poor physiological function and disease risk.^26^ While reduced fetal growth is associated with restrictive lung deficits, prematurity is associated with more obstruction.^27^ These findings support public health interventions to improve the average birth weight to optimize lung function at a population level. Some interventions and preventive measures, particularly targeting high risk populations, have been proposed for example, improvement of women's nutritional status^28^ and smoking cessation interventions during pregnancy.^29^ However, studies evaluating whether attempts to enhance intra‐uterine growth have a long‐lasting influence on lung health are needed.

The main strength of this study is that the TAHS is the only long‐term respiratory cohort with birth data that has measured complex lung function in middle‐age. Findings on static lung volumes provide the best available evidence that low birth weight is truly linked to restrictive lung deficits. While most previous studies did not disentangle the relative importance of low birth weight and gestational age,^12^ we were able to investigate the independent association for birth weight using SGA as the primary exposure and adjusting for gestational age when birth weight was the outcome. Moreover, most of birth weight data (66%) were ascertained from public hospital birth records. Findings were substantially unchanged when the analyses excluded birth weight determined by parental recall.

While this analysis has been based on a subset of the original whole‐of‐population TAHS cohort, selection bias related to missing information is unlikely to have been present as those with or without birth weight data had similar baseline characteristics, including lung function at 7 years. Furthermore, additional adjustment from sample weights yielded similar findings. However, a limitation is that smoking status during pregnancy was self‐reported by the parents, thus might be susceptible to reporting error, though our information on this seems typical for the era. The use of parental SES when the participants were 7 years as a proxy for SES during pregnancy is a limitation, but any changes of SES were likely to be non‐differential. Finally, although we were able to adjust for different prenatal factors, there is still the possibility that other unmeasured factors including genetic differences may contribute to the association between birth weight and adult lung function.

In conclusion, this study found that being SGA was associated with a lower FEV_1_, FVC, D_L_co, RV and TLC in middle age, and these associations were largely mediated by adult height. This suggests that the long‐term detrimental effect of reduced fetal lung growth is associated with lower adult lung function which is most likely due to smaller lungs with little evidence of any specific parenchymal impairment. These findings highlight the importance of preventive strategies that aim to reduce factors that predispose to SGA.

## AUTHOR CONTRIBUTION

**Melvin Tandra:** Conceptualization (lead); data curation (lead); formal analysis (lead); methodology (equal); writing – original draft (lead); writing – review and editing (lead). **E. Haydn Walters:** Conceptualization (supporting); funding acquisition (equal); investigation (equal); methodology (equal); supervision (supporting); writing – original draft (supporting); writing – review and editing (equal). **Jennifer Perret:** Conceptualization (supporting); formal analysis (supporting); funding acquisition (supporting); investigation (supporting); methodology (equal); supervision (supporting); writing – original draft (supporting); writing – review and editing (equal). **Adrian J. Lowe:** Data curation (supporting); formal analysis (supporting); funding acquisition (supporting); investigation (supporting); methodology (equal); writing – original draft (supporting); writing – review and editing (equal). **Caroline J. Lodge:** Data curation (supporting); formal analysis (supporting); funding acquisition (supporting); investigation (supporting); methodology (equal); writing – original draft (supporting); writing – review and editing (equal). **David P. Johns:** Data curation (supporting); funding acquisition (equal); investigation (equal); methodology (equal); writing – original draft (supporting); writing – review and editing (equal). **Paul S. Thomas:** Data curation (supporting); funding acquisition (equal); investigation (equal); methodology (equal); writing – original draft (supporting); writing – review and editing (equal). **Gayan Bowatte:** Data curation (equal); methodology (equal); writing – original draft (supporting); writing – review and editing (equal). **Peter G. Davis:** Conceptualization (supporting); formal analysis (supporting); investigation (supporting); methodology (supporting); writing – original draft (supporting); writing – review and editing (supporting). **Michael J. Abramson:** Conceptualization (supporting); formal analysis (supporting); funding acquisition (equal); investigation (equal); methodology (equal); supervision (supporting); writing – original draft (supporting); writing – review and editing (equal). **Shyamali C. Dharmage:** Conceptualization (equal); formal analysis (equal); funding acquisition (lead); investigation (lead); methodology (lead); supervision (equal); writing – original draft (supporting); writing – review and editing (equal). **Dinh S. Bui:** Conceptualization (equal); data curation (equal); formal analysis (equal); investigation (equal); methodology (equal); supervision (lead); writing – original draft (equal); writing – review and editing (equal).

## CONFLICTS OF INTEREST

Michael Abramson received grants from Pfizer. Sanofi, GSK and Boehringer Ingelheim outside the submitted work. Other authors had not disclosed any conflicts of interest. Caroline Lodge, E. Haydn Walters, Adrian Lowe, Dinh Bui, Michael Abramson, Jennifer Perret ad Shyamali Dharmage hold an investigator‐initiated grant from GlaxoSmithKline for unrelated research. Adrian Lowe has received non‐financial support from Primus Pharmaceuticals for unrelated research.

## HUMAN ETHICS APPROVAL DECLARATION

The study was approved by the Human Ethics Review Committees of all relevant institutions. Written informed consent was obtained from all participants.

## Supporting information


**Appendix S1** Additional Methods.

**Visual Abstract** Association between lung function and birth weight

## Data Availability

Individual participant data may be provided on request by anyone with a proposal. The proposal will be considered by the TAHS steering committee. Request can be directed to SCD (who is PI of TAHS and corresponding author of this article). Data for all TAHS participants may be provided.
