# Supplementary material for: Small for gestational age is associated with reduced lung function in middle age: A prospective study from first to fifth decade of life
Source: Respirology. 2022 Oct 5;28(2):159–65. doi: 10.1111/resp.14379 (PMC10947040; doi:10.1111/resp.14379)
Supplement: Supplementary file 1 — Appendix S1 Additional Methods. [file RESP-28-159-s002.docx]

**SUPPORTING INFORMATION**

**Small for gestational age is associated with restrictive lung function deficits in middle age: A prospective study from 1^st^ to 5^th^ decade of life**

Tandra M^1^, Walters EH ^1,2^, Perret J^1^, Lowe AJ^1^, Lodge CJ^1^, Johns DP^2^, Thomas PS^3^, Bowatte G^1,4^, Davis PG^5^, Abramson MJ6^6^, Dharmage SC^1*^, Bui DS^1*^

**Appendix S1-** Additional Methods

**Data collection**

Data on birthweight and gestational age were extracted from birth records. We obtained all available hospital birth records for children born in 1961 in Tasmania from the Tasmanian State Archive and from Tasmanian hospitals (2775 participants). Birthweight data were available from the state archive for the Queen Alexandra Hospital for Women (Hobart), Beaconsfield District Hospital, Ouse District Hospital, Ulverstone District Hospital, Mersey General Hospital, Toosey Memorial Hospital at Langford and Sheffield District Nursing. Additional records were obtained from Launceston General Hospital and North West General Hospital. Parents provided birth weight information for 2265 participants, of whom 859 also had birth weight from hospital records. Thus, 4181 participants had information on birth weight (2775 from hospital records and additional 1406 from parental report).

**Definition of variables**.

BMI was defined as the weight in kg divided by height in meters squared.

**Figure S1** *–* The Directed Acyclic Graph (DAG)


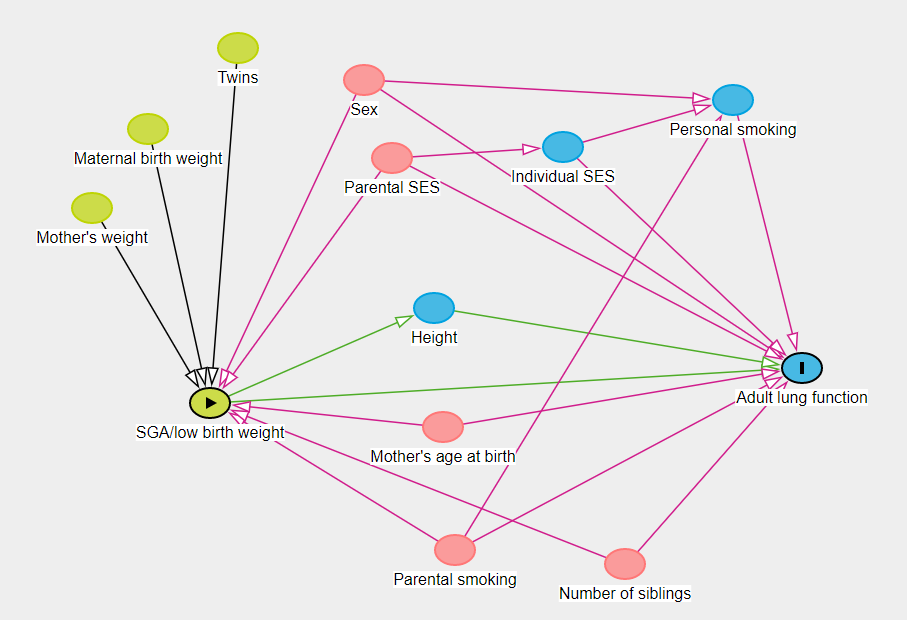


**Table S1.** Characteristics at 7 years among those with or without birth weight data.

| Characteristics | Participants with birth weight data (N=4181) | Participants without birth weight data (N=4402) | P-value |
| --- | --- | --- | --- |
| Male sex, % | 51.2% | 51.2% | 0.96 |
| Maternal age at birth, mean (SD) years | 27.2(8.6) | 27.5(6.9) | 0.09 |
| Maternal smoking | 65.4% | 58.8% | <0.001 |
| Paternal Smoking | 40.9% | 36.1% | <0.001 |
| Childhood asthma | 17.3% | 16.3% | 0.20 |
| Childhood lung function  FEV_1_, % predicted, mean (SD)  FVC, % predicted, mean (SD)  FEV_1_/FVC, %, mean (SD) | 99.2 (12.8)  97.5 (12.5)  90.9 (5.8) | 98.7(17.5)  97.0(18.5)  90.7 (5.9) | 0.12  0.20  0.22 |
| Childhood SES  1 (highest)  2  3  4  5 | 21.3%  6.5%  30.7%  28.6%  12.8% | 20.5%  6.3%  28.6%  28.4%  16.1% | 0.15 |
| Childhood pneumonia | 13.9% | 14.7% | 0.26 |

**Table S2.** Characteristics of participants with or without SGA

| **Characteristics** | **Small for gestational age group** | **Appropriate for gestational age group** |
| --- | --- | --- |
| Male sex, % | 57.2% | 53.0% |
| Smoking status, %  Never  Past  Current | 41.6%  32.8%  25.6% | 45.2%  29.5%  25.3% |
| BMI at 45 years, kg/m^2^, mean (±SD) | 28.9 ± 6.3 | 28.3 ± 6.5 |
| Height at 45 years, cm, mean (±SD) | 168.3 ± 8.0 | 171.2 ± 8.6*** |
| Current asthma, % | 24.1% | 22.3% |
| Usual cough | 23.3% | 20.8% |
| Usual phlegm | 18.1% | 20.5% |
| Wheezing last year | 39.8% | 30.9%* |
| Nocturnal chest tightness | 22.4% | 20.0% |
| Nocturnal shortness of breath | 12.4% | 11.8% |
| Shortness of breath when  hurrying on level ground | 15.2% | 13.9% |
| Post-BD FEV1, L, mean (±SD) | 3.32 ± 0.67 | 3.48 ± 0.68* |
| Post-BD FVC, L, mean (±SD) | 4.26 ± 0.87 | 4.44 ± 0.88* |
| Post-BD TLC, L, mean (±SD) | 6.29 ± 1.2 | 6.56 ± 1.2* |
| Maternal smoking during pregnancy, % | 26.8% | 18.8% |
| Paternal smoking during pregnancy, % | 66.3% | 61.6% |
| Maternal age at birth, years, mean (SD) | 25.9 ± 4.6 | 26.3 ± 5.5 |

*p<0.05; **p<0.01; ***p<0.001

**Table S3.** Adjusted associations between birth weight and pre-bronchodilator lung function at 45 years

|  | Small for Gestational Age (binary variable)  *Mean Difference (95% CI)* ǁ | Birthweight allowing for GA  *Mean Difference (95% CI) per kg* ƚ |
| --- | --- | --- |
| FEV_1_, mL | **-177 (-298 to 57)**** | **113 (30 to 196)**** |
| FVC, mL | **-197 (-344 to 50)**** | **123 (22 to 224)***** |
| FEV_1_/FVC ratio, % | -0.51(-2.17 to 1.14) | 0.22 (-0.92 to 1.36) |

ƚ adjusted for sex, age, age of mother at birth, parental smoking, number of siblings, parental social class, as well as gestational age

ǁ adjusted for sex, age, age of mother at birth, parental smoking, number of siblings and parental social class

*p<0.05; **p<0.01; ***p<0.001

**Table S4.** Adjusted associations between birth weight only collected from hospital records and post-bronchodilator lung function at 45 years

|  | Small for Gestational Age (binary variable)  *Mean Difference (95% CI)* ǁ | Birthweight allowing for GA  *Mean Difference (95% CI) per kg* ƚ |
| --- | --- | --- |
| FEV_1_, mL | -123 (-258 to 11) | **124 (19 to 230)*** |
| FVC, mL | **-195 (-358 to -32)*** | **163 (36 to 291)*** |
| FEV_1_/FVC ratio, % | 0.42 (-1.50 to 2.35) | -0.40 (-1.92 to 1.12) |
| TLC, mL | **-371 (-624 to -118)**** | **211 (16 to 406)*** |
| RV, mL | **-174 (-361 to -33)*** | 59 (-49 to 169) |
| D_L_co, mmol/min/kPa | -0.29 (-0.77 to 0.19) | 0.35(-0.02 to 0.72) |
| K_CO_, mmol/min/kPa/L | 0.02 (-0.05 to 0.09) | -0.003 (-0.06 to 0.05) |
| FRC, mL | -161 (-369 to 46) | 63 (-96 to 223) |

ƚ adjusted for sex, age, age of mother at birth, parental smoking, number of siblings, parental social class, as well as gestational age

ǁ adjusted for sex, age, age of mother at birth, parental smoking, number of siblings and parental social class

*p<0.05; **p<0.01; ***p<0.001

**Table S5.** Adjusted associations of SGA and birth weight with z-scores of post-bronchodilator lung function at 45 years

|  | Small for Gestational Age (binary variable)  *Mean Difference (95% CI)* ǁ | Birthweight adjusted for GA  *Mean Difference (95% CI) per kg* ƚ |
| --- | --- | --- |
| FEV_1_, z-score | -0.14 (-0.34, 0.071) | 0.035 (-0.11, 0.18) |
| FVC, z-score | -0.043 (-0.23, 0.14) | -0.010 (-0.13, 0.13) |
| FEV_1_/FVC, z-score | -0.13 (-0.34, 0.077) | 0.046 (-0.10, 0.19) |
| TLC, z-score | -0.11 (-0.28, 0.079) | 0.054 (-0.075, 0.18) |
| RV, z-score | -0.12 (-0.30,0.060) | 0.027 (-0.10, 0.15) |
| FRC, z-score | -0.094 (-0.28, 0.091) | 0.029 (-0.10, 0.16) |
| D_L_co, z-score ¶ | 0.073 (-0.15, 0.30) | 0.093 (-0.071, 0.25) |
| K_CO_, z-score | -0.029 (-0.26, 0.21) | 0.050 (-0.11, 0.22) |

ƚ adjusted for age of mother at birth, parental smoking, number of siblings, parental social class, as well as gestational age

ǁ adjusted for age of mother at birth, parental smoking, number of siblings and parental social class

¶ values were adjusted to a standard haemoglobin concentration and corrected for the presence of carboxyhaemoglobin.

*p<0.05; **p<0.01; ***p<0.001

**Table S6** – Mediation analysis for childhood height (at 7 years) as a mediator of the associations of small for gestational age and birth weight with lung function at 45 years.

| Outcomes | Indirect effect (ACME) (95%CI) | Direct effect (95%CI) | % mediated |
| --- | --- | --- | --- |
|  | **Association with small for gestational age** | | |
| FEV_1_ | -0.025 (-0.051 to -0.005) | -0.181 (-0.286 to -0.064) | 12.1% |
| FVC | -0.043 (-0.083 to -0.008) | -0.177 (-0.304 to -0.037) | 19.6% |
| TLC | -0.066 (-0.129 to -0.012) | -0.277 (-0.476 to -0.056) | 19.3% |
| D_L_co | -0.086 (-0.180 to -0.011) | -0.361 (-0.706 to 0.018) | 19.0% |
|  | **Association with birth weight** | | |
| FEV_1_ | 0.028 (0.014 to 0.050) | 0.091 (0.008 to 0.171) | 23.4% |
| FVC | 0.048 (0.023 to 0.081) | 0.075 (0.024 to 0.172) | 39.0% |
| TLC | 0.066 (0.028 to 0.115) | 0.143 (0.011 to 0.291) | 31.8% |
| D_L_co | 0.091 (0.034 to 0.166) | 0.268 (0.001 to 0.524) | 25.2% |

ACME = Average Causal Mediation Effects

**Visual Abstract** Association between lung function and birth weight
