# Supplementary material for: Small for gestational age is associated with reduced lung function in middle age: A prospective study from first to fifth decade of life
Source: Respirology. 2022 Oct 5;28(2):159–65. doi: 10.1111/resp.14379 (PMC10947040; doi:10.1111/resp.14379)
Supplement: Supplementary file 2 — Visual Abstract Association between lung function and birth weight [file RESP-28-159-s001.pdf]

Association between lung function and birth weight

For every one kilogram of birth weight:

- FEV1 increased by **117** mL
- FVC increased by **124** mL
- TLC increased by **215** mL
- DLCO increased by 0.36 mmol/min/kPa

Small for gestational age babies, compared to their normal peers, had, at 45 years old:

- **191** mL reduced FEV1
- **205** mL reduced FVC
- **292** mL reduced TLC
- **126** mL reduced RV
- **0.42** mmol/min/kPa reduced DLCO

***In both cases*:**

- There was ***no significant change, or comparable*** FEV1/FVC ratios.
- These associations were **significantly mediated** by adult height (56% - 90%)

Respirology

Small for gestational age is associated with reduced lung function in middle age: A prospective study from 1st to 5th decade of life - Tandra et al. *Respirology* 2022; DOI: 10.1111/resp.14379
